# Metagenomic sequencing reveals viral diversity of mosquitoes from Shandong Province, China

**DOI:** 10.1128/spectrum.03932-23

**Published:** 2024-03-11

**Authors:** Yuhao Wang, Xiaojuan Lin, Chao Li, Guifang Liu, Suting Wang, Meng Chen, Xuemin Wei, Hongling Wen, Zexin Tao, Yifei Xu

**Affiliations:** 1 Department of Microbiology, School of Public Health, Cheeloo College of Medicine, Shandong University, Jinan, Shandong, China; 2 Division of EPI, Shandong Center for Disease Control and Prevention, Jinan, Shandong, China; 3 Suzhou Research Institute of Shandong University, Suzhou, Jiangsu, China; State Key Laboratory of Virology, School of Public Health, Wuhan University, Wuhan, China

**Keywords:** metagenomic sequencing, virome, mosquito-borne virus, novel virus

## Abstract

Mosquitoes carry a large number of known and unknown viruses, some of which could cause serious diseases in humans or animals. Metagenomic sequencing for mosquito viromes is crucial for understanding the evolutionary history of viruses and preventing emerging mosquito-borne diseases. We collected 1,598 mosquitoes belonging to four species from five counties in Shandong Province, China in 2021. They were grouped by species and sampling locations and subjected to metagenomic next-generation sequencing for the analysis of the viromes. A total of 233,317,352 sequencing reads were classified into 30 viral families and an unclassified group. Comparative analysis showed that mosquitoes in Shandong Province generally possessed host-specific virome. We detected mosquito-borne viruses including Japanese encephalitis virus, Getah virus, and Kadipiro virus in *Culex tritaeniorhynchus* and *Anopheles sinensis* samples. Phylogenetic analysis showed that these pathogenic viruses may have existed in mosquitoes in Shandong Province for a long time. Meanwhile, we identified 22 novel viruses belonging to seven families and the genus *Negevirus*. Our study comprehensively described the viromes of several common mosquito species in Shandong Province, China, and demonstrated the major role of host species in shaping mosquito viromes. Furthermore, the metagenomic data provided valuable epidemiological information on multiple mosquito-borne viruses, highlighting the potential risk of infection transmission.

**IMPORTANCE:**

Mosquitoes are known as the source of various pathogens for humans and animals. *Culex tritaeniorhynchus*, *Armigeres subalbatus*, and *Anopheles sinensis* have been found to transmit the Getah virus, which has recently caused increasing infections in China. *Cx. tritaeniorhynchus* and *Culex pipiens* are the main vectors of Japanese encephalitis virus and have caused epidemics of Japanese encephalitis in China in past decades. These mosquitoes are widely present in Shandong Province, China, leading to a great threat to public health and the breeding industry. This study provided a comprehensive insight into the viromes of several common mosquito species in Shandong Province, China. The metagenomic sequencing data revealed the risks of multiple pathogenic mosquito-borne viruses, including Japanese encephalitis virus, Getah virus, and Kadipiro virus, which are of great importance for preventing emerging viral epidemics.

## INTRODUCTION

Mosquitoes were known as transmitting vectors of many arboviruses, including but not limited to Japanese encephalitis virus (JEV), West Nile virus, and dengue virus, which can infect humans and animals through biting (1–3). The zoonotic transmission of these arboviruses has caused public health concerns and enormous economic burdens worldwide for a long time (4–6). Most of the known arboviruses are RNA viruses, which possess greater genetic diversity compared with DNA viruses. The long-term mosquito-virus coevolution led to a wide range of animal hosts and increasing transmission patterns of these viruses (7). Some invertebrate viruses in mosquitoes, while cannot infect humans or animals directly, could modulate the transmission of pathogenic viruses (8, 9). Thus, surveillance for viral populations in mosquitoes is critical for the prevention and control of emerging mosquito-borne viruses.

The rapid development of next-generation sequencing technology enabled the non-specific detection of viromes in mosquitoes and promoted the surveillance for viromes and diseases (10). Metagenomic investigation of mosquito viromes has been conducted in many regions around the world, not only focusing on mosquito-borne viruses but also attempting to profile the entire viral community in mosquitoes (11–13). However, the differences in mosquito species and geographic regions made it challenging to establish universal results through regional research. Although there is a lack of global system analysis, some studies have confirmed that mosquito viromes were closely related to host species and the ecological environment (14, 15). Both the host’s biological differences and ecological barriers may significantly limit the virus transmission among mosquitoes. This characteristic highlights the necessity of performing regional and personalized surveillance. In addition, research on mosquito viromes usually comes with the discovery of many novel viruses, especially insect-specific viruses. The high proportion of novel viruses also indicates the ongoing need to comprehensively understand the diversity of mosquito-associated viruses (16, 17).

Shandong Province is located in eastern China and is the second most populous province in the country. The wide distribution of wetlands makes it a suitable breeding ground for many kinds of common mosquitoes in China. However, few investigations have been conducted for mosquito virome in this region.

Here, we describe metagenomic sequencing for viromes of four common mosquito species, including *Anopheles sinensis*, *Armigeres subalbatus*, *Culex pipiens*, and *Culex tritaeniorhynchus* from Shandong Province, China. We assessed the viral compositions of different mosquito species and compared the effects of host and geographic factors on viral diversity. We detected, annotated, and performed phylogenetic analysis for multiple pathogenic viruses against animals and 22 novel viruses. This study provided baseline information for the surveillance of mosquito-associated viruses.

## MATERIALS AND METHODS

### Sample collection

A total of 1,598 mosquitoes were collected from five counties across Shandong Province in the summer of 2021. All mosquitoes were collected at the local pig farm using handheld mosquito aspirators and zapper lamps. These pig farms have diverse and dense populations of mosquitoes and are more likely to become breeding grounds for some zoonotic mosquito-borne viruses such as JEV. Mosquitoes were classified morphologically into four groups including *Ar. subalbatus*, *An. sinensis*, *Cx. tritaeniorhynchus*, and *Cx. pipiens*, based on the characteristics of wings, abdominal patterns, antennae, and scales (18). These mosquitoes were grouped into 10 pools for sequencing based on species and sampling locations. The number of mosquitoes in each pool ranged from 43 to 225.

### Sample processing and metagenomic sequencing

Mosquito pools were homogenized in a mixer mill MM400 (Retsch GmbH, Germany) for 10 min at 20/s after the addition of 1 mL of MEM (Gibco, USA) and three 3 mm steel balls to each tube. After centrifugation at 12,000 × *g* for 30 min, the supernatant was forwarded to viral RNA extraction using MagMAX Pathogen RNA/DNA Kit (Thermo Fisher, Lithuania). The ribosomal RNA was removed using Ribo-off rRNA Depletion Kit (Bacteria; Vazyme, #N407). The prokaryotic RNA library was prepared using MGIEasy RNA directional library preparation kit V2.0 (16 RXN). The 100 bp paired-end sequencing was performed on DNBSEQ (DNBSEQ Technology) platform.

### Viral contig assembly and annotation

Quality control was performed for the raw sequencing reads using Fastp v0.22.0 (19). Reads with an average quality of less than 30 and bases with a quality of less than 20 at both ends were removed. Host reads were removed by mapping to mosquito genomes (*Ar. subalbatus*, GenBank accession number GCA_024139115.1; *An. sinensis*, GCA_000441895.2; *Cx. quinquefasciatus*, GCF_000209185.1; *Cx. pipiens*, GCF_016801865.1) with bwa v0.7.17 (20). Sequencing reads for the *Cx. tritaeniorhynchus* pools were mapped to a *Cx. quinquefasciatus* reference genome because there was no publicly available *Cx. tritaeniorhynchus* genome. The resulting reads were *de novo* assembled into contigs using the MEGAHIT v1.2.9 with default parameters (21). The contigs were then compared to the non-redundant protein (nr) database using the DIAMOND Blastx program v2.0.13.151 with an E-value cutoff of 1E−5 (22). Contigs were identified as putative viral sequences if their top Blast hit was annotated under the “Viruses” superkingdom. These putative viral contigs were further classified into viral species by aligning with the non-redundant nucleotides (nt) database and non-redundant protein (nr) database using Blastx and Blastn (23). Mammal-associated viruses were identified based on the host categories annotated in National Center for Biotechnology Information (https://www.ncbi.nlm.nih.gov/).

To identify contigs of novel viruses, open reading frames (ORFs) in putative viral sequences were predicted using ORFfinder (https://www.ncbi.nlm.nih.gov/orffinder/) with the minimal length of 100 amino acids (aa) and further compared against the Conserved Domain Database using Batch Conserved Domain Search tools (https://www.ncbi.nlm.nih.gov/Structure/bwrpsb/bwrpsb.cgi). Contigs encoding RNA-dependent RNA polymerase (RdRp) domains were retained. The contigs considered to represent the (nearly) complete viral genome or coding sequence were selected according to Blastx results. Consensus sequences were generated by mapping non-host reads to corresponding contigs or reference sequences. Novel virus species were recognized according to the species demarcation criteria recommended by the International Committee on Taxonomy of Viruses (ICTV; https://talk.ictvonline.org/ accessed on February 2023) based on the nt and aa sequence identities. If clear criteria were not available for a genus in ICTV, the criterion that <90% aa identity of the RdRp domain or <80% nt identity across the whole genome with known viruses was applied. The novel viruses were named “Shandong mosquito virus” with their family information and an order number attached. The consensus sequences of novel viruses were annotated using Geneious v2022.2.2 (24).

### Quantification of virus abundance

Two approaches were applied to determine viral abundance at the family and species levels. At the family level, the non-host reads were classified using kraken2 v2.0.7 (25). A custom database including all “virus” sequences in nt database, standard kraken2 database, and novel virus sequences identified in this study was used. The viral reads identified by kraken2 were subjected to diamond Blastx comparisons against nr databases to exclude false positives (the top Blast hit was not under the “Viruses” superkingdom). The abundance of each viral family was quantified as the number of identified reads per million (RPM) total filtered reads. Only viral families over one RPM were retained. At the species level, the non-host reads were mapped to selected reference genomes from GenBank or assembled viral contigs. The mapped reads of each viral species were counted and further quantified as RPM. A viral species was considered present if it had at least 10 mapped reads. A set of high-abundance viruses was selected for comparative analysis of viral compositions.

### Comparative analysis of viral compositions

Comparative analysis was performed at the family and species levels. Visualization of viral abundance and relevant hierarchical clustering analyses was performed in R 4.13 with ggpolt2 and pheatmap packages (26). Principal coordinates analysis (PCoA) based on Bray–Curtis distance was performed with a vegan package (27).

### Phylogenetic analysis

Phylogenetic analyses were performed for viruses identified in this study with corresponding reference sequences from GenBank. Sequence alignment was performed using MAFFT v7.310 (28). Phylogenetic trees were built using IQ-TREE v2.0.3 with branch support by ultrafast bootstrap approximation test (29). The phylogenetic trees were visualized using R package ggtree v3.2.1 (30).

### JEV-specific real-time PCR assay

Viral nucleic acids were extracted from 200 µL of mosquito grinding supernatant using virus DNA and RNA extraction kit (Tianlong, Xian, China). The JEV-specific primers and probes for reverse transcription-PCR were designed as previously described (31). Quantitative reverse transcriptase-PCR (qRT-PCR) assay for JEV was performed using ABI AgPath-ID One-Step RT-PCR Reagents kit.

## RESULTS

### Metagenomic sequencing of mosquitoes in Shandong

**Fig 1:**
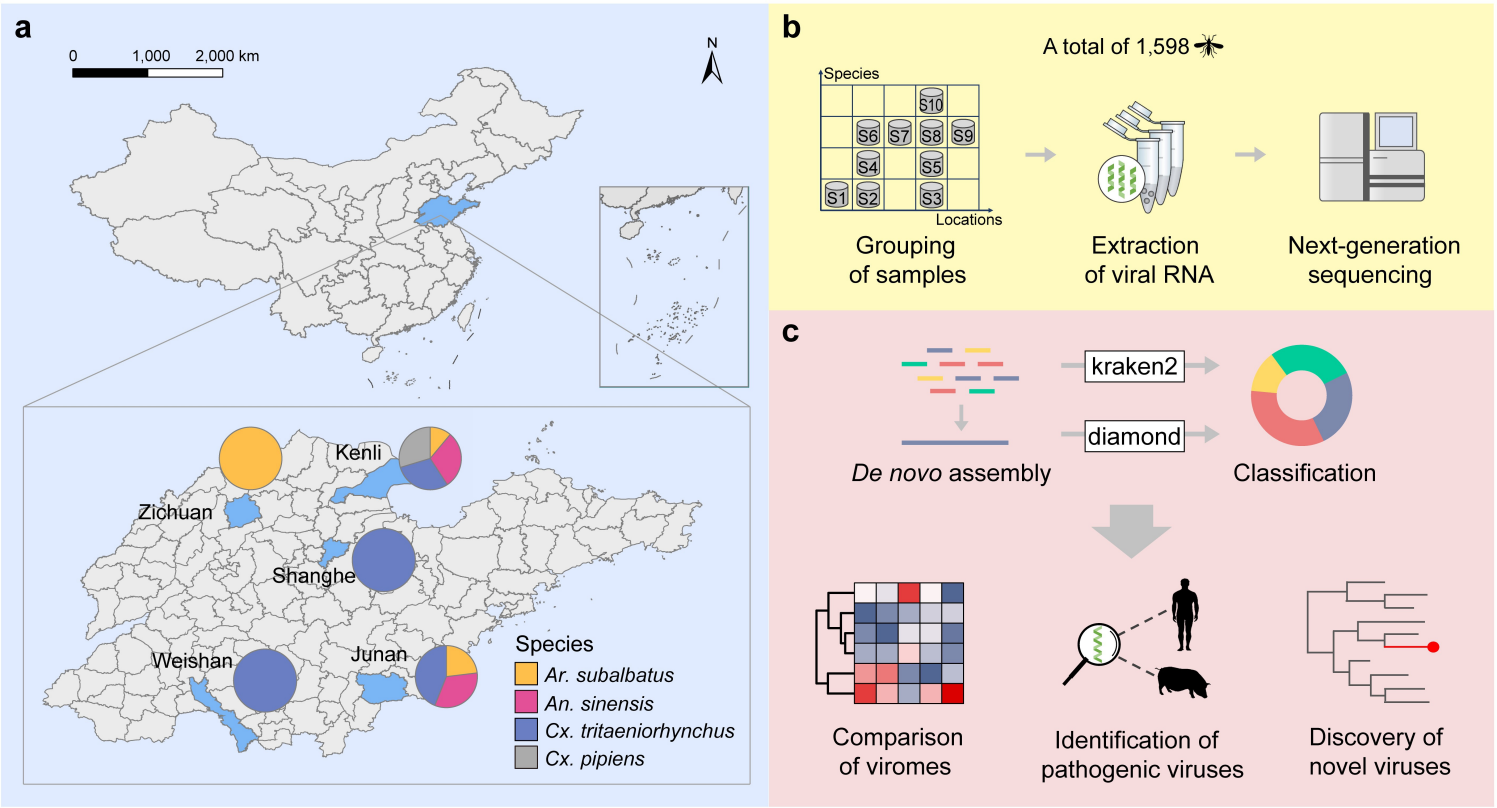
Overview of sampling, metagenomic sequencing, and viromics analysis of mosquitoes from Shandong. (**a**) Geographic map of sampling of mosquitoes in Shandong. (**b**) Pooling and metagenomic sequencing of mosquito samples. (**c**) Bioinformatics analysis framework for mosquito viromes.

We collected 1,598 mosquitoes belonging to four species (*An. sinensis*, *Ar. subalbatus*, *Cx. tritaeniorhynchus*, and *Cx. pipiens*) in five counties of Shandong Province, China (Fig. 1a). We grouped mosquitoes into 10 pools according to species and sampling locations (Table 1; Fig. 1b). Metagenomic sequencing generated 12,012,990,120 paired-end reads for the 10 pools. After quality control and removal of host sequences, we retained 2,835,821,270 clean reads for downstream analysis (Fig. 1c). We identified a total of 233,317,352 viral reads belonging to 30 viral families and unclassified viruses, accounting for 8.23% of the total clean reads.

**TABLE 1:** Biological classification, sampling locations, and pooling information of mosquitoes collected from five counties in Shandong Province, China

Mosquito genus	Mosquito species	Pool name	Location	Average altitude	Date (yr/mo/day)	Climate	No. of mosquitoes
*Armigeres*	*Ar. subalbatus*	S1	Zichuan	185	2021/8/19	Rain	43
		S2	Junan	200	2021/8/18	Sunny	105
		S3	Kenli	6	2021/8/24–2021/8/25	Sunny	75
*Anopheles*	*An. sinensis*	S4	Junan	200	2021/8/18	Sunny	150
		S5	Kenli	6	2021/8/24–2021/8/25	Sunny	200
*Culex*	*Cx. tritaeniorhynchus*	S6	Junan	200	2021/8/18	Sunny	200
		S7	Weishan	32	2021/8/16–2021/8/17	Sunny	200
		S8	Kenli	6	2021/8/24–2021/8/25	Sunny	200
		S9	Shanghe	16	2021/8/24	Sunny	225
	*Cx. pipiens pallens*	S10	Kenli	6	2021/8/24–2021/8/25	Sunny	200

### Virome profiles of different mosquito species

**Fig 2:**
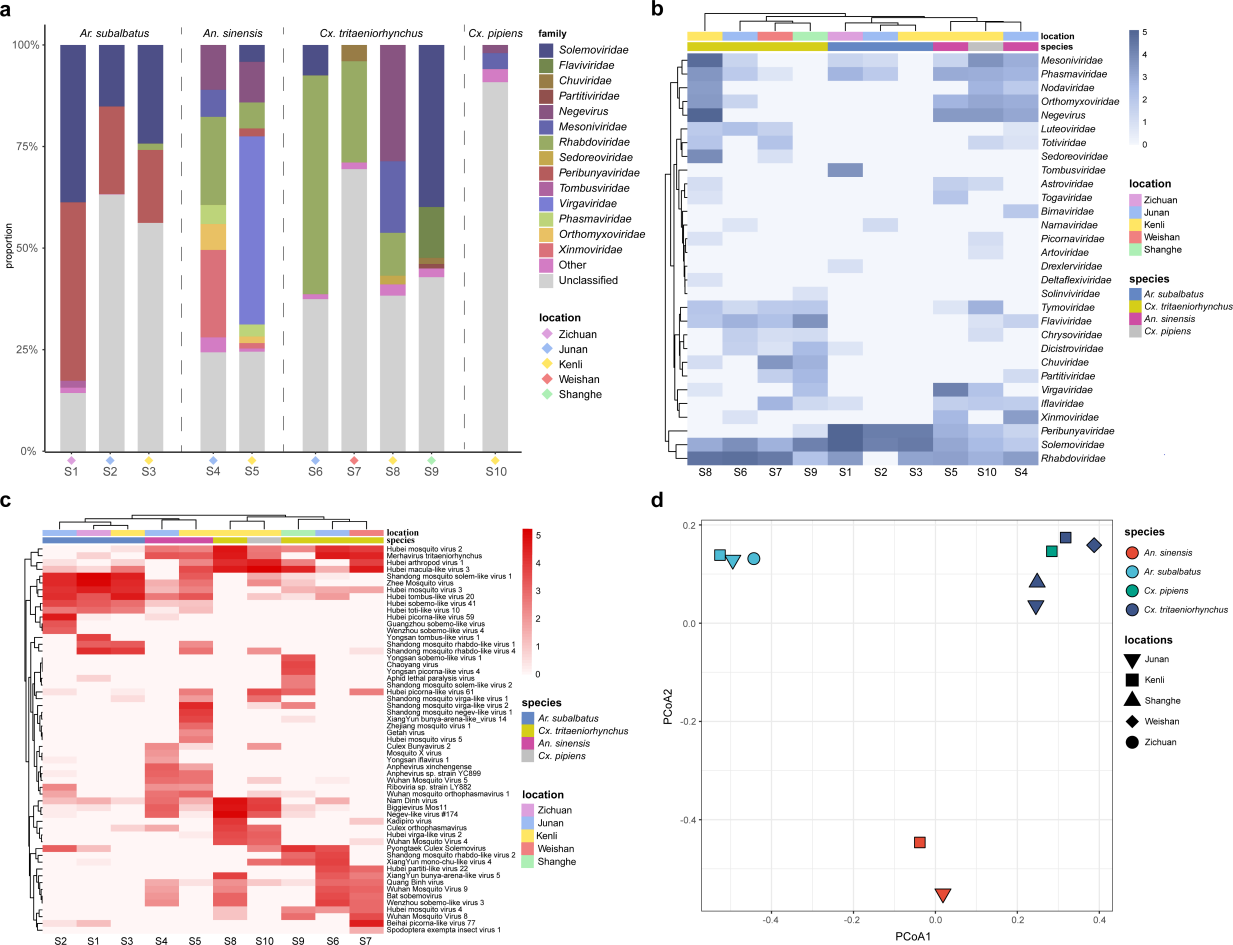
Comparison of mosquito viromes between mosquito species and locations at the family and species levels. (**a**) Proportions of viral families among all classified viral reads. Families with a proportion lower than 1% are grouped as “others.” Heat map of abundance for viral families (**b**) and species (**c**). The cells show the base-10 logarithm of the normalized abundance of each viral family. The hierarchical clustering of the columns is based on the Euclidean distance. The location and species of the mosquitoes were marked with different colors. (**d**) PCoA for the viral compositions at species level among 10 samples. PERMANOVA test on mosquito species: *P* < 0.001 and *R*^2^ = 0.565.

In three *Ar. subalbatus* samples (S1, S2, and S3), we identified a large proportion of reads from families *Peribunyaviridae* (18.0%–43.9% of viral reads) and *Solemoviridae* (15.1%–38.7% of viral reads; Fig. 2a). Analysis at the species level showed that these reads mainly belonged to Zhee Mosquito virus and a newly identified novel virus Shandong mosquito solem-like virus 1.

*Cx. tritaeniorhynchus* samples (S6, S7, S8, and S9) possessed different viromes. In samples S6 and S7, we found high abundances of family *Rhabdoviridae* (24.8%–53.9% of viral reads) mainly from Merhavirus tritaeniorhynchus (Fig. 2a). The other two *Cx. tritaeniorhynchus* samples (S8 and S9) possessed high proportions of family *Mesoniviridae* (17.6% of viral reads in S8), *Solemoviridae* (39.9% of viral reads in S9), and genus *Negevirus* (28.6% of viral reads in S8).

In two *An. sinensis* samples (S4 and S5), *Xinmoviridae* (21.49% of viral reads in S4) and *Virgaviridae* (46.29% of viral reads in S5) were the most abundant viral families (Fig. 2a). The newly identified virus Shandong mosquito virga-like virus 2 accounted for a high proportion in sample S5. In addition, both two samples (S4 and S5) processed many reads from Merhavirus tritaeniorhynchus, which was also present in *Cx. tritaeniorhynchus* samples (S6 and S8) from the same sampling site.

The only *Cx. pipiens* sample (S10) showed a unique viral family composition, with most of the reads not being classified to the family level.

We observed a large proportion of unclassified viruses (viruses without family annotation) at both the family and species levels (Fig. 2b and c). Among them, Hubei tombus-like virus 20 (in samples S1, S2, S3, and S5), Hubei macula-like virus 3 (in samples S5, S7, S8, S9, and S10), and Negev-like virus #174 (in samples S8 and S10) were present in multiple samples with high abundance. Additionally, there were abundant viral reads belonging to Hubei picorna-like virus 59 and XiangYun bunya-arena-like_virus 14 in samples S2 and S5, respectively.

### Comparison and diversity of mosquito viromes

At the family level, the hierarchical clustering result showed that 10 samples were generally clustered by mosquito species. *Ar. subalbatus* samples (S1, S2, and S3) and *Cx. tritaeniorhynchus* samples (S6, S7, and S9) clustered according to host species, while a *Cx. tritaeniorhynchus* sample S8 formed a separate group. The *Cx. pipiens* sample (S10) clustered together with *An. sinensis* samples (S4 and S5), with only a few reads (less than 10% of viral read), was successfully classified into families.

The species-level analysis including unclassified viruses showed a more clear clustering by mosquito species rather than sampling sites (Fig. 2c). The *Cx. pipiens* sample (S10) clustered closely with *Cx. tritaeniorhynchus* samples (S6, S7, S8, and S9), which were from the same mosquito genus. The PCoA result further supported the clustering by mosquito species (Fig. 2c). The *Ar. subalbatus* samples showed smaller internal Bray–Curtis distances compared to *Cx. tritaeniorhynchus* and *An. sinensis* samples.

### Mammal-associated viruses in mosquito

We identified several pathogenic mammal-associated viruses. Among these, we recovered two complete genomic sequences. We performed phylogenetic analysis to investigate the potential risk of mosquito-borne diseases.

**Fig 3:**
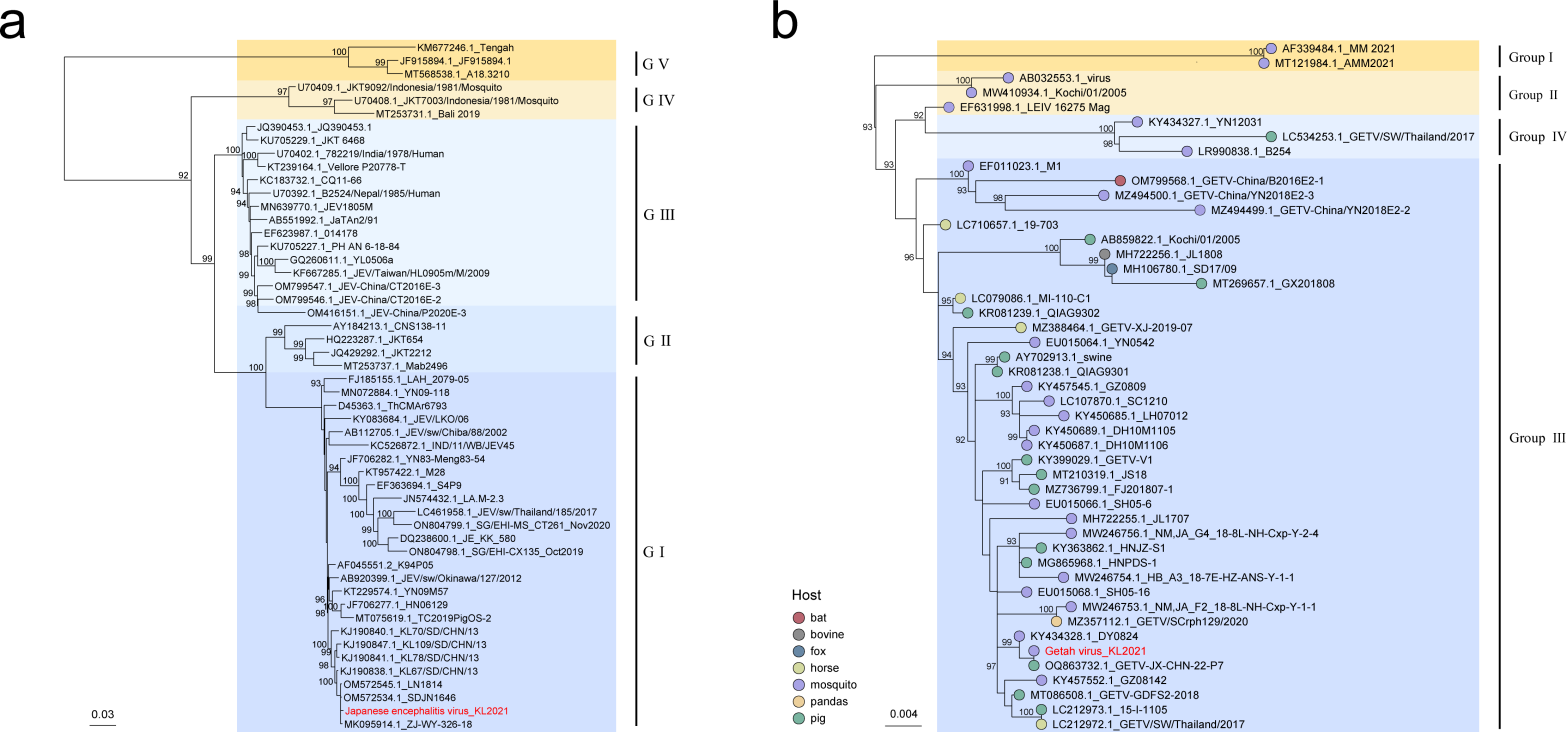
Maximum likelihood phylogenetic analysis for mosquito-borne viruses detected from mosquito samples in Shandong Province, China. (**a**) Phylogenetic tree of JEV based on the nucleotide sequence of envelope gene. (**b**) Phylogenetic tree of Getah virus (GETV) based on the nucleotide sequence of E2 gene. Sequences recovered in this study are displayed in red. Colored dots represent the host species. Numbers at the nodes indicate ultrafast bootstrap support evaluated by 1,000 replicates; only bootstrap values above 90 are shown.

We identified 460 reads (2.48 RPM) from JEV in a *Cx. tritaeniorhynchus* sample (S8) and recovered an incomplete genome sequence with >80% coverage of the E gene. The E gene-specific phylogeny showed that our JEV sequence fell into a Shandong lineage clade within the genotype I group (G I), which is the dominant genotype in China (Fig. 3a) (32). The qRT-PCR assay further confirmed the existence of JEV G I with cycle threshold (Ct) values of 30.5. Blast results showed that our incomplete E gene sequence shared high similarity (97%–99%) with multiple JEV strains from this Shandong lineage.

Getah virus (GETV) is a mosquito-borne virus that can infect a wide range of mammals including humans. In an *An. sinensis* sample (S5), we detected 23,636 GETV (178.95 RPM) reads. We recovered a complete GETV genome and named it “GETV strain KL2021.” The E2 gene-specific phylogeny showed that GETV can be classified into four genetic groups, and our sequence was in group III (Fig. 3a). Our E2 gene sequence shared 99.84% nucleotide identity with an E2 gene fragment (GenBank accession no. KY434328) from local mosquitoes 13 years ago. Additionally, some closely related GETV strains to our sequence were present in other provinces of China in recent years. The most related strain is GETV-JX-CHN-22-P7 (GenBank accession no. OQ863732.1), which shared 100% nucleotide identity of the E2 gene with our sequence.

Kadipiro virus (KDV) is a 12-segmented RNA virus first isolated from mosquitoes and proved to possess tropism for humans (33). There were only three full genomic sequences of KDV in GenBank (accessed on July 2023). We detected KDV reads in four *Cx. tritaeniorhynchus* samples (S6, S7, S8, S9) and recovered a full genomic sequence from sample S8. Blast results showed that all 12 segments of our KDV sequences shared high nucleotide identities (96.41%–99.80%) with KDV strain SDKL1625 from *An. sinensis* from the same county in 2016 (GenBank accession no. MG590140-MG590151) (34). Although KDV was regionally prevalent in two common mosquito species, no infection to vertebrates was observed.

We detected some viruses transmitting in mammals without the involvement of arthropod vectors (e.g., papillomavirus, Sapporo virus, and some viruses in the genus *Mamastrovirus*; Table 2). These viruses were most likely to originate from human/animal blood or tissue ingested by mosquitoes. Blast results showed that the contigs of Sapporo virus and Mamastrovirus were closely related to multiple pig-associated strains, suggesting potential infections in local pig farms.

**TABLE 2:** Summary of mammal-associated viruses identified in various mosquito species from Shandong using metagenomic next-generation sequencing

Mosquito species	Collection location	Virus	Reads	Abundance (RPM)	Coverage (%)
*An. sinensis*	Kenli	GETV	23,636	178.95	99.62
		*Mamastrovirus^a^* (Porcine astrovirus)	2,212	16.75	52.08
		Sapporo virus^*a*^	15	0.11	24.29
*Cx. tritaeniorhynchus*	Junan	Gammapapillomavirus 12^*a*^	363	1.45	92.98
		Mupapillomavirus 2^*a*^	385	1.53	84.06
		KDV	16	0.06	4.38
	Weishan	KDV	1,453	9.94	89.52
	Kenli	KDV	1,948,445	10,515.45	100.00
		JEV	460	2.48	69.41
	Shanghe	KDV	448	0.46	47.6
*Cx. pipiens*	Kenli	*Mamastrovirus^a^* (Porcine astrovirus)	2,235	8.12	54.72
		Sapporo virus^*a*^	452	1.64	55.32

^*a*^ Viruses transmitting in mammals without the participation of arthropod vectors.

### Annotation and phylogenetic analysis of novel viruses

We identified 22 novel viruses for which (nearly) complete genome or complete coding regions of RdRp were recovered (Table S1). They were classified into seven viral families and the genus *Negevirus*.

#### Rhabdoviridae

**Fig 4:**
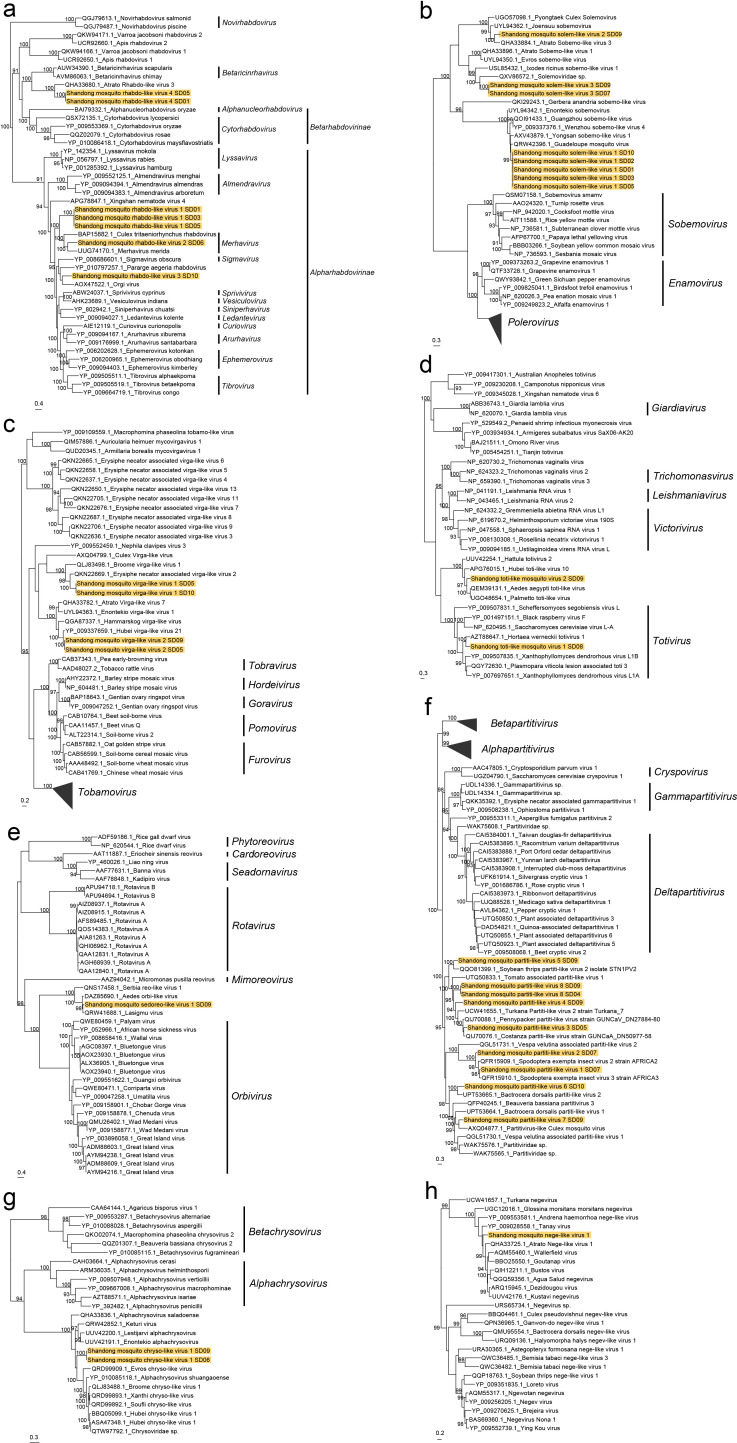
Phylogenetic analysis for novel viruses from seven families identified by metagenomic next-generation sequencing for mosquitoes in Shandong Province, China. (**a**) Phylogenetic trees for aa sequence of L protein of *Rhabdoviridae*. (**b**) Phylogenetic trees for aa sequence of RdRp of *Solemoviridae*. (**c**) Phylogenetic trees for aa sequence of replication protein of *Virgaviridae*. (**d**) Phylogenetic trees for aa sequence of RdRp of *Totiviridae*. (**e**) Phylogenetic trees for aa sequence of RdRp of *Sedoreoviridae*. (**f**) Phylogenetic trees for aa sequence of RdRp of *Partitiviridae*. (**g**) Phylogenetic trees for aa sequence of RdRp of *Chrysoviridae*. (**h**) Phylogenetic trees for aa sequence of RdRp of *Negevirus*. Sequences discovered in this study are marked with yellow backgrounds. Numbers at the nodes indicate ultrafast bootstrap support evaluated by 1,000 replicates; only bootstrap values above 90 are shown.

We identified four novel viruses belonging to the family *Rhabdoviridae* (Shandong mosquito rhabdo-like virus 1–4), which could infect vertebrates, invertebrates, and plants (35). The novel virus Shandong mosquito rhabdo-like virus 1 was most closely related to the Merida virus (49% aa identity of L protein). The L protein-specific phylogeny showed that Shandong mosquito rhabdo-like virus 1 formed a new branch close to genus Merhavirus clade. (Fig. 4a) Shandong mosquito rhabdo-like virus 2 showed 73% aa identity of L protein to Formosus virus, clustered in the clade of genus Merhavirus, in which some viruses can infect mammalian cell lines in vitro (36). The L protein sequence of Shandong mosquito rhabdo-like virus 3 showed 47% aa identity to that of Orgi virus, phylogenetically falling into an unclassified branch within the clade of subfamily *Alpharhabdovirinae*. Shandong mosquito rhabdo-like virus 4 was most related to San Gabriel mononegavirus (52%–53% aa identity of L protein) and separated from any known genera or subfamilies in the phylogenetic tree.

#### Solemoviridae

We identified three novel viruses (Shandong mosquito solem-like virus 1–3) with bisegmented genomes in the family *Solemoviridae*. Although *Solemoviridae* was previously considered a monopartite viral family, there were many bisegmented Sobemo-like viruses newly classified into this family (37, 38). We identified both segments of Shandong mosquito solem-like virus 1 in three mosquito species, with a high abundance in *Ar. Subalbatus* (Fig. 2b). The RdRp sequence of this virus shared more than 80% aa identity with several Sobemo-like viruses found in mosquitoes (Enontekio sobemovirus, Guadeloupe mosquito virus, etc.). Novel viruses Shandong mosquito solem-like virus 2–3 were most similar to Atrato Sobemo-like virus 3 (72% aa identity) and Solemoviridae sp. isolate YSN675 (53%–54% aa identity), respectively. These two novel viruses were inferred to be bisegmented according to their related viruses, though only the RdRp coding segments were detected. The RdRp-specific phylogeny showed that all three novel viruses clustered together with a series of Sobemo-like viruses (Fig. 4b).

**Fig 5:**
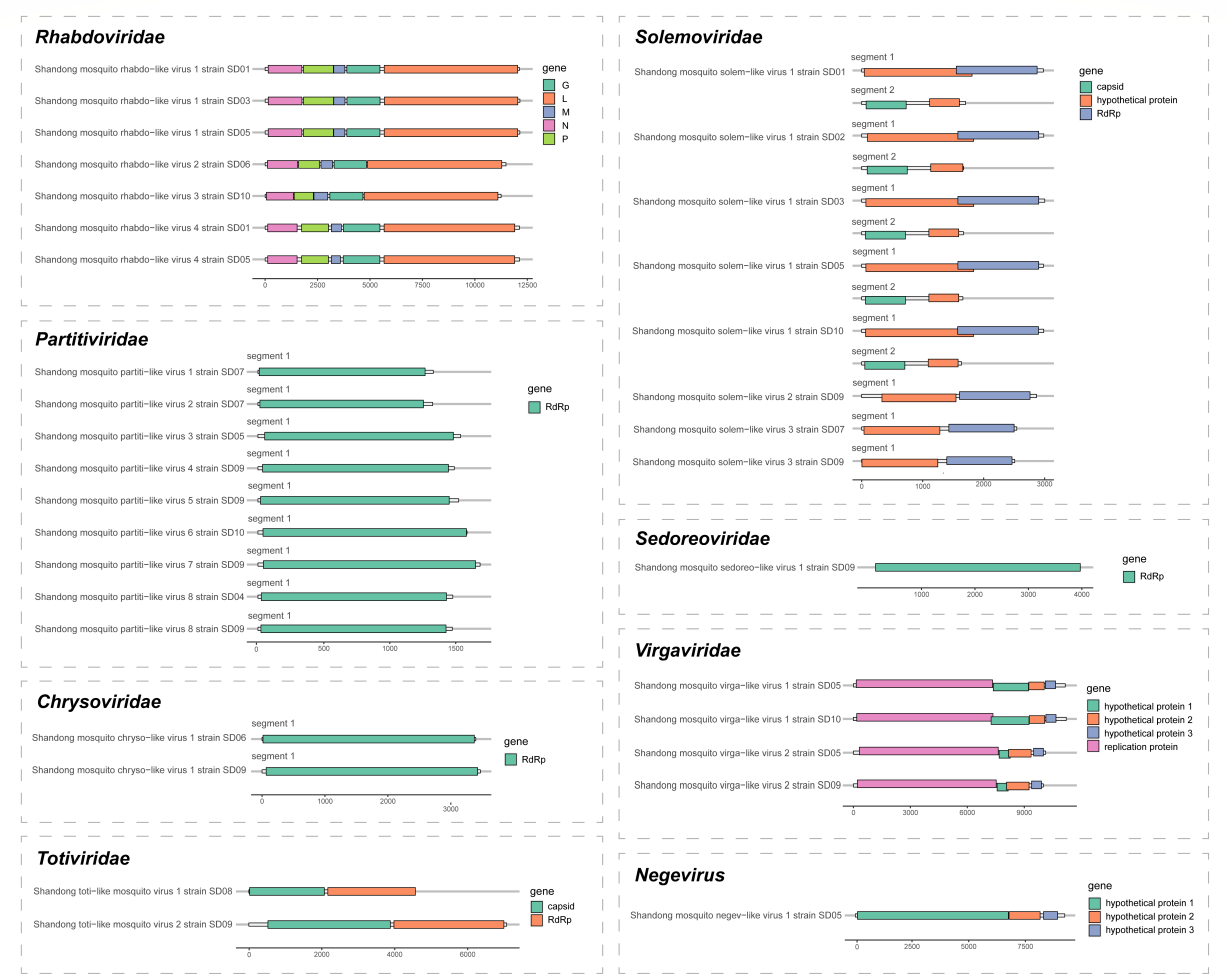
Genome organizations of 22 novel viruses identified from metagenomic next-generation sequencing from mosquitoes in Shandong Province, China.

#### Virgaviridae

We identified two new novel viral species belonging to the family *Virgaviridae* (Shandong mosquito virga-like virus 1–2). The novel virus Shandong mosquito virga-like virus 1 was most related to Erysiphe necator-associated virga-like virus 2 (55% aa identity of replication protein) and approximately 2,400 bp longer in genome (Fig. 5). The exceeded part of Shandong mosquito virga-like virus 1 was most similar to Hubei virga-like virus 18 strain fly97447. The other novel virus Shandong mosquito virga-like virus 2 showed 79% aa identity of replication protein to Hubei virga-like virus 21. Both novel viruses were phylogenetically clustered with many unclassified *Virgaviridae* viruses (Fig. 4c).

#### Totiviridae

We identified two novel viral species (Shandong mosquito toti-like virus 1–2) belonging to the family *Totiviridae*. The RdRp sequence of Shandong mosquito toti-like virus 1 was most related to Hortaea werneckii totivirus 1 (57% aa identity). Phylogenetic analysis showed that Shandong mosquito toti-like virus 1 clustered within the clade of genus *Totivirus*. The RdRp sequence of Shandong mosquito toti-like virus 2 was most related to Aedes aegypti toti-like virus (60% aa identity) and fell into an unclassified clade close to the genus *Totivirus* (Fig. 4d).

#### Sedoreoviridae

We identified a novel virus species (Shandong mosquito Sedoreo-like virus 1) in the family *Sedoreoviridae*, which infects a wide range of hosts including mammals (39). This novel virus shared 89% RdRp aa identity to Lasigmu virus, forming a new cluster near the root of the genus *Orbivirus* together with some unclassified viruses identified from arthropods (Fig. 4e).

#### Partitiviridae

*Partitiviridae* was traditionally associated with fungi, plants, and protozoa, with bisegmented genomes encoding RdRP and coat protein, respectively (40). We detected nine genome segments of RdRp related to the family *Partitiviridae*, which belongs to eight novel species (Shandong mosquito Partiti-like virus 1–8). These viruses were mainly related to a series of unclassified viruses of *Partitiviridae* isolated from arthropods. The RdRp-specific phylogeny showed that these arthropod-associated unclassified viruses formed a single cluster separated from established genera, suggesting a new genus with a distinct host range (Fig. 4f).

#### Chrysoviridae

We identified a novel virus species (Shandong mosquito chryso-like virus 1) belonging to the family *Chrysoviridae* based on the RdRp segments obtained in *Cx. tritaeniorhynchus*. The RdRp sequences were most related to Lestijarvi alphachrysovirus (62%–64% aa identity). Phylogenetic analysis showed that it clustered with several unclassified *Chrysoviridae* viruses separated from the established genera (Fig. 4g).

#### Negevirus

We identified a novel virus, Shandong mosquito negev-like virus 1, belonging to the genus *Negevirus*, a newly established taxon that had not been officially accepted by ICTV (41). This novel virus was most related to Tanay virus (74% aa identity of RdRp domain). RdRp-specific phylogeny showed that the Shandong mosquito negev-like virus 1 clustered with a group of mosquito-associated viruses of *Negevirus* (Fig. 4h).

## DISCUSSION

We profiled the viromes for four common mosquito species from Shandong Province, China, at the family and species levels. Our data showed that mosquito viromes were dominated by a small number of extremely abundant virus species. *Rhabdoviridae*, *Solemoviridae*, *Phasmaviridae*, *Peribunyaviridae*, and *Mesoniviridae* were the most prevalent viral families from four mosquito species. Meanwhile, we observed a large proportion of viruses that had not been classified into established families in some samples. Most of these viruses were discovered in arthropods by metagenomic next-generation sequencing and were considered to be arthropod-specific (38).

We identified several high-abundance viruses related to pathogenic arboviruses, such as Merhavirus tritaeniorhynchus in family *Rhabdoviridae*, Zhee Mosquito virus in family *Peribunyaviridae*, and Chaoyang virus and Quang Binh virus in family *Flaviviridae*. Previous studies found that Merhavirus tritaeniorhynchus can infect mammalian cell lines *in vitro*, suggesting the potential cross-species transmission of these viruses (36). Interestingly, we observed plant-associated viral families such as *Solemoviridae* in all the samples. This result suggested that mosquitoes may act as transmission vectors for plant viruses by mechanical transmission or other potential mechanisms. Furthermore, we identified some viruses (Nam Dinh virus and Biggievirus Mos11) previously detected from mosquitoes in other continents (42). This finding suggested the global transmission capacity and wide host permissiveness of these viruses.

Comparative analysis across different mosquito species and sampling locations deepened our understanding of the diversity of mosquito viromes. Our data at the family and species levels showed that the mosquito viromes were mainly determined by host factors within a specific geographical range. Analysis for classified families showed host-specific viral compositions within *Ar. subalbatus* and *An. sinensis*, while the viromes of *Cx. tritaeniorhynchus* varied greatly with sampling locations. When analyzing at the species level and considering the unclassified viruses without family information, we observed a more clear clustering by mosquito classification. These results suggested that some unclassified viruses were likely more species-specific components in the viromes of *Cx. tritaeniorhynchus*. Notably, the *Cx. pipiens* sample shared a similar virome with *Cx. tritaeniorhynchus*. This indicated that mosquito viromes may be genus-specific rather than species-specific, consistent with the pattern found in the viromes of ticks (43). Another interesting finding is that *Ar. subalbatus* showed a smaller internal Bray–Curtis distance than *Cx. tritaeniorhynchus* at the species levels. This result may indicate that *Ar. subalbatus* possessed more consistent viromes, while the viromes of *Cx. tritaeniorhynchus* were more sensitive to geographical factors. The sensitiveness to geographical factors of virome may vary with mosquito species, influenced by the mosquito’s living habits and the long-distance spreading capability of corresponding viruses. A larger geographical span may intensify the geographical impact on host-specific viromes, leading to diverse viral populations even for the same mosquito species (44). Future work with a larger sampling scale and quantity is required to more comprehensively understand the viral diversity in mosquitoes.

We identified multiple pathogenic arboviruses including JEV, GETV, and KDV. We recovered two complete genome sequences for GETV and KDV from metagenomic next-generation sequencing data. These viruses were closely related to locally prevalent genetic lineages and were believed to have existed for a long time in the local zoonotic transmission cycle. Our data suggested that the GETV may have spread among local mosquitoes for 13 years. Recently, a more closely related GETV sequence was detected from another province in China, indicating possible interregional transmissions. Moreover, the incomplete E gene of JEV obtained in *Cx. tritaeniorhynchus* showed high homology with the strains detected from *Cx. tritaeniorhynchus* in Shandong 10 years ago. This Shandong JEV lineage was believed to be associated with a JEV outbreak in Shandong in 2013 with 407 cases and 11 deaths (45). It is necessary to implement continuous surveillance to generate information for the prevention and control of such outbreaks in the future. In addition, we unexpectedly detected many incomplete sequences of pathogenic vertebrate viruses, suggesting the potential role of mosquitoes as a sentinel model to survey virus burden in animal farms (12). Overall, our data provided baseline information for local mosquito-associated pathogenic viruses and demonstrated the great benefits that can be gained from metagenomic sequencing for the surveillance of arboviruses.

There are some limitations to our study. First, we did not clean up the blood in mosquito bodies before pooling. Therefore, we detected some vertebrate viruses that were likely originated from animal or human blood/tissue. Second, our mosquitoes were collected from pig farms, which may give them specific food sources and microbial communities. A previous study observed that habitat type influenced the compositions of mosquito viromes (15). The single habitat type may limit the representativeness of our samples. In addition, considering the small size and large population of mosquitoes, we pooled mosquito individuals for each sample. Thereby, the results could be strongly influenced by a small number of mosquito individuals carrying abundant viruses. New sequencing approaches using single mosquitoes could contribute to a more precise understanding of the mosquito viromes (46).

Our study characterized the viromes of common mosquito species from Shandong, China. These mosquitoes generally possessed host-specific viromes in Shandong Province. Furthermore, our finding provided molecular epidemiological information for multiple mosquito-borne viruses. These findings demonstrated the great application value of metagenomic sequencing for the surveillance of mosquito-associated viruses.

## Data Availability

The sequencing data produced in this study have been deposited in the NCBI Sequence Read Archive (SRA) database under the BioProject accession number PRJNA1080457.

## SUPPLEMENTAL MATERIAL

Table S1 spectrum.03932-23-s0001.xlsx Name, classification, completeness, and related reference sequence of each novel virus identified in this study.

Table S2 spectrum.03932-23-s0002.xlsx Overview of viral species identified in each sample and corresponding reads mapping to selected reference sequences.

ASM does not own the copyrights to Supplemental Material that may be linked to, or accessed through, an article. The authors have granted ASM a non-exclusive, world-wide license to publish the Supplemental Material files. Please contact the corresponding author directly for reuse.
